# High-energy, Long-cycle-life Secondary Battery with Electrochemically Pre-doped Silicon Anode

**DOI:** 10.1038/s41598-020-59913-4

**Published:** 2020-02-21

**Authors:** Ying Wang, Masaharu Satoh, Masazumi Arao, Masashi Matsumoto, Hideto Imai, Hiroshi Nishihara

**Affiliations:** 1 Department of Chemistry, School of Science, The University of Tokyo, 7-3-1 Hongo, Bunkyo-ku, Tokyo 113-0033 Japan; 2 Analysis Platform Department, NISSAN ARC, LTD., 1 Natsushima-cho, Yokosuka, 237-0061 Japan

**Keywords:** Batteries, Batteries

## Abstract

Electrochemical pre-doping of a silicon electrode was investigated to create a new class of rechargeable battery with higher energy density. The electrochemical reaction during pre-doping formed a high-quality solid electrolyte interface (SEI) on the surface of silicon particles, which improved the charge and discharge cycle life with a small irreversible capacity. The surface composition of the pre-doped silicon particles was characterized using transmission electron microscopy (TEM), solid state magic-angle-spinning (MAS) nuclear magnetic resonance (NMR) and X-ray diffraction analysis (XRD). Pressurization promoted SEI growth and lithium binding with silicon to form Li_15_Si_4_ accompanied by the reductive reaction product of Li_2_CO_3_ originated from electrolyte. The Li_15_Si_4_ was highly stable when the silicon anode was used in a full cell, thus resulting in a silicon anode with a long cycle life.

## Introduction

An increasing number of intelligent devices, electric vehicles, and smart energy management systems are being used in all parts of society, and this growing market, especially in mobility and transportation, requires low-cost rechargeable batteries with high energy density. Rechargeable lithium-ion batteries (LIBs) are integral to these sophisticated devices; however, a serious drawback is that their energy density and  capacity density have not increased substantially^1^. To solve this problem, silicon is an attractive anode material for the next generation of LIBs due to its high capacity of 3572 mA h/g^2^, which is approximately 10 times larger than that of graphite (372 mA h/g).

In the reaction mechanism of silicon during charging and discharging, the phase transition from the crystalline to amorphous state proceeds via the formation of a lithiated amorphous silicide in the initial discharging stage, followed by the formation of Li_15_Si_4_ in the deep discharging stage^2^.

The high capacity of silicon causes a large expansion of electrode volume of up to 300–400%, leading to the collapse of fine particle structures, and thus causing a dramatic decline in cycle performance. Various types of silicon nanoparticles^3–5^ and nanowires^6,7^ have shown longer cycling lives than micron-sized particles. The large surface area of nanostructured silicon should produce irreversible capacity caused by the formation of a solid electrolyte interface (SEI) passivation layer^8^. The SEI is important in silicon anodes because it allows the steady intercalation and desorption of lithium through the surface of the silicon particles. The SEI provides an inactivated and stabilized conductive pathway for lithium ions and not for electrons, and is even impenetrable to the electrolyte, which prevents further growth of the SEI^9,10^.

The formation of a stabilized SEI is still challenging because in conventional pre-doping methods, such as contact or electrochemical pre-doping, some of the lithium doping forms the SEI on the surface of silicon particles, and the rest of the lithium intercalates into the silicon particles. The intercalation causes the expansion and collapse of the silicon particles, continuously exposing new surfaces where the SEI is formed. This process destroys the silicon particles completely before the SEI layer is fully formed on their surfaces. Two main solutions to overcome these problems have been proposed. The first is using a binder, particularly durable binders, such as polyimides^11^ or styrene-butadiene rubber^12^ to maintain the size of silicon particles, but the method requires complicated pretreatments. The second is limiting the voltage range during battery operation to keep the volume change small, which improves the cycle life at the expense of the capacity^13–15^. These methods are unsuitable for industrial-scale production and remain at the laboratory scale.

This study proposes a pre-doping process for silicon anodes, in which electrochemical pre-doping under pressure produces the SEI on the surface of silicon particles. The electrochemical reaction during pre-doping with electrolyte additives produces a high-quality SEI. Furthermore, the pressure relaxes the transformation strain of the silicon anode during pre-doping in a controlled manner that avoids the collapse of the silicon fine structure. We describe the properties and structure of a silicon anode electrochemically pre-doped under pressure.

## Electrochemical Pre-Doping under Pressure

**Figure 1:**
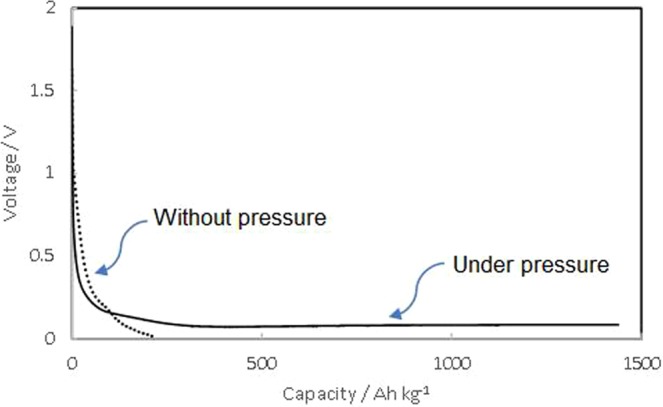
Electrochemical profiles of the lithium ion pre-doping of silicon measured at a constant current value of 0.11 mA/cm^2^ under pressure and without pressure.

Figure 1 shows the voltage vs. capacity profiles for the lithium pre-doping for silicon electrodes at current density of 0.11 mA h/cm^2^ (125 mA h/g-Si) under pressure and without pressure. Electrochemical pre-doping without pressure terminated earlier with a notable voltage drop, leaving SEI formation unfinished. In contrast, pre-doping under pressure continued to a capacity of more than 1500 A h/kg. This indicates that the internal resistance was reduced under pressure, which may relate to the change in electrode thickness in the cells. Actually, the thickness was decreased from 25 µm to 18 µm (28%) by applying the pressure of 50 kPa. Furthermore, pre-doping under pressure was expected to prevent the transformation and detachment of the electrode, improving the stability and charge and discharge cyclability.

The charge capacity of the pre-doped silicon anode in the first cycle shows the amount of intercalated and removable free lithium besides that consumed to form the SEI. The electric charge consumed for SEI formation was quantified by the total charge during the pre-doping process and in the first charging process. The cycle performance indicated the quality of the SEI formed in the pre-doping process.

**Figure 2:**
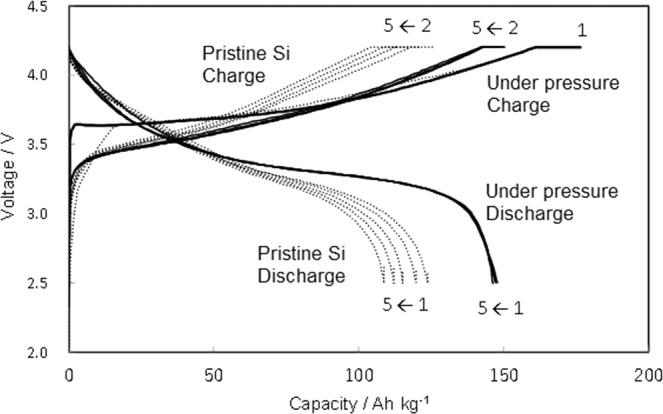
Charge and discharge curves of a silicon anode electrochemically pre-doped under pressure and a pristine silicon anode in LiNCM cathode cells for the first five cycles.

Figure 2 shows the charge and discharge curves of full cells containing either a silicon anode electrochemically pre-doped under pressure or a pristine silicon anode with a LiNCM cathode for the first five cycles. The anode/cathode balance of the full cells was set to 1.0 assuming that apparent capacity of silicon is 2500 mA h/g-Si by adjusting the amount of silicon on anode and that of LiNCM on cathode. The capacity of the cell containing a pre-doped silicon anode was consistent with the theoretical value for LiNCM of 150 A h/kg. In contrast, the LiNCM electrode paired with a pristine silicon anode lost 15% of its capacity because the charging of silicon is not reversible. Moreover, the capacity decreased considerably as the number of cycles increased.

**Figure 3:**
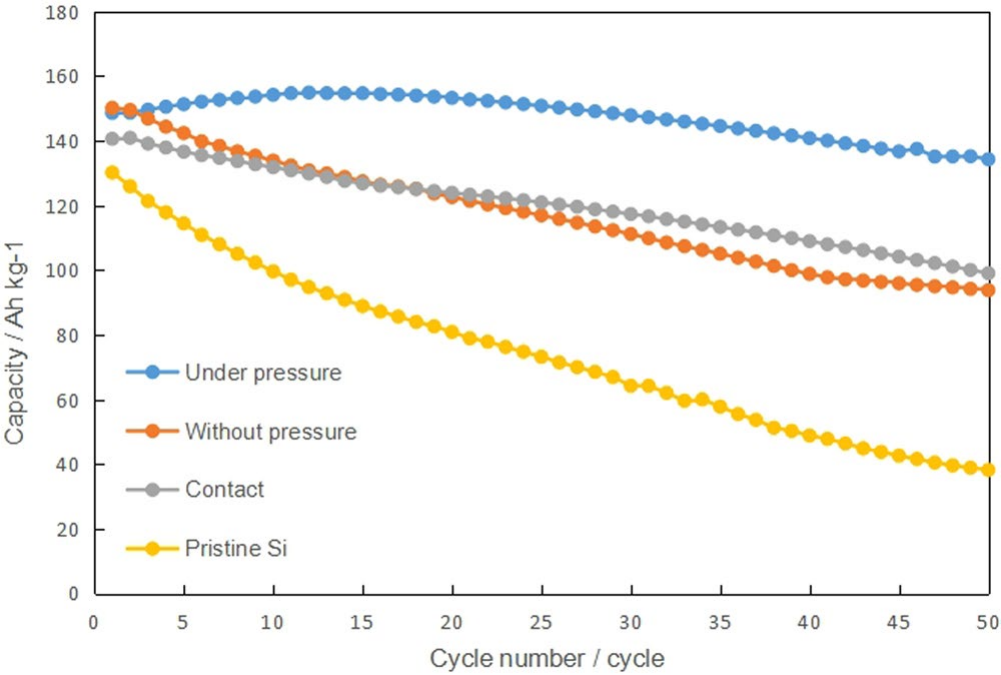
Cycle performance of full cells with a LiNCM cathode and various silicon anodes of electrochemically pre-doped under pressure, electrochemically pre-doped without pressure, contact pre-doped with lithium metal, and pristine silicon.

Figure 3 shows the capacity as a function of cycle number for full cells containing a LiNCM cathode and a silicon anode electrochemically pre-doped under pressure or without pressure. As a reference, the results for full cells containing a LiNCM cathode with contact pre-doped or pristine silicon anodes are shown. The pristine sample degraded within 20 cycles and formed neither an SEI nor pre-doped lithium in advance, meaning that lithium was consumed from the cathode leads to poor round Coulomb efficiency during the cycles. A linear decrease in capacity was observed, which may have been caused by the binder retaining the particle structure; however, the structure collapsed. In the contact pre-doped sample, the decrease in capacity was smaller than for the pristine sample due to the lithium pre-doping, and degradation was inevitable without SEI formation.

The sum of the lithium in anode and cathode is an important issue in this study. The amount of lithium in silicon anodes electrochemically pre-doped under pressure and without pressure, and contact pre-doped are 466, 140, and 1150 mA h/g-Si, respectively, which are considerably lower than the theoretical capacity of silicon, 3572 mA h/g-Si. Extra lithium provided from pre-doped silicon anode is partly consumed for SEI formation effective for long-term cycling stability. These results demonstrate that our pre-doping method produced superior electrochemical properties to overcome traditional barrier about poor round Coulomb efficiency of silicon anode during cycles.

## Characterization of Pre-Doped Silicon Particles

We investigated how the pre-doping methods affected the structures and the electrochemical properties of silicon. The electrochemically pre-doped samples prepared under pressure and without pressure were analyzed by TEM to examine structural and surface composition changes as a function of the pressure. If the current is small, the silicon electrode can be pre-doped to a higher lithium concentration. We obtained pre-doped silicon samples with a Li/Si ratio of 2 mol/mol by electrochemical pre-doping at a current value of 0.044 mA/cm^2^ without pressure. For both samples under pressure and without pressure, the capacity density of silicon anode pre-doping was set to 2.2 mA h/cm^2^.

**Figure 4:**
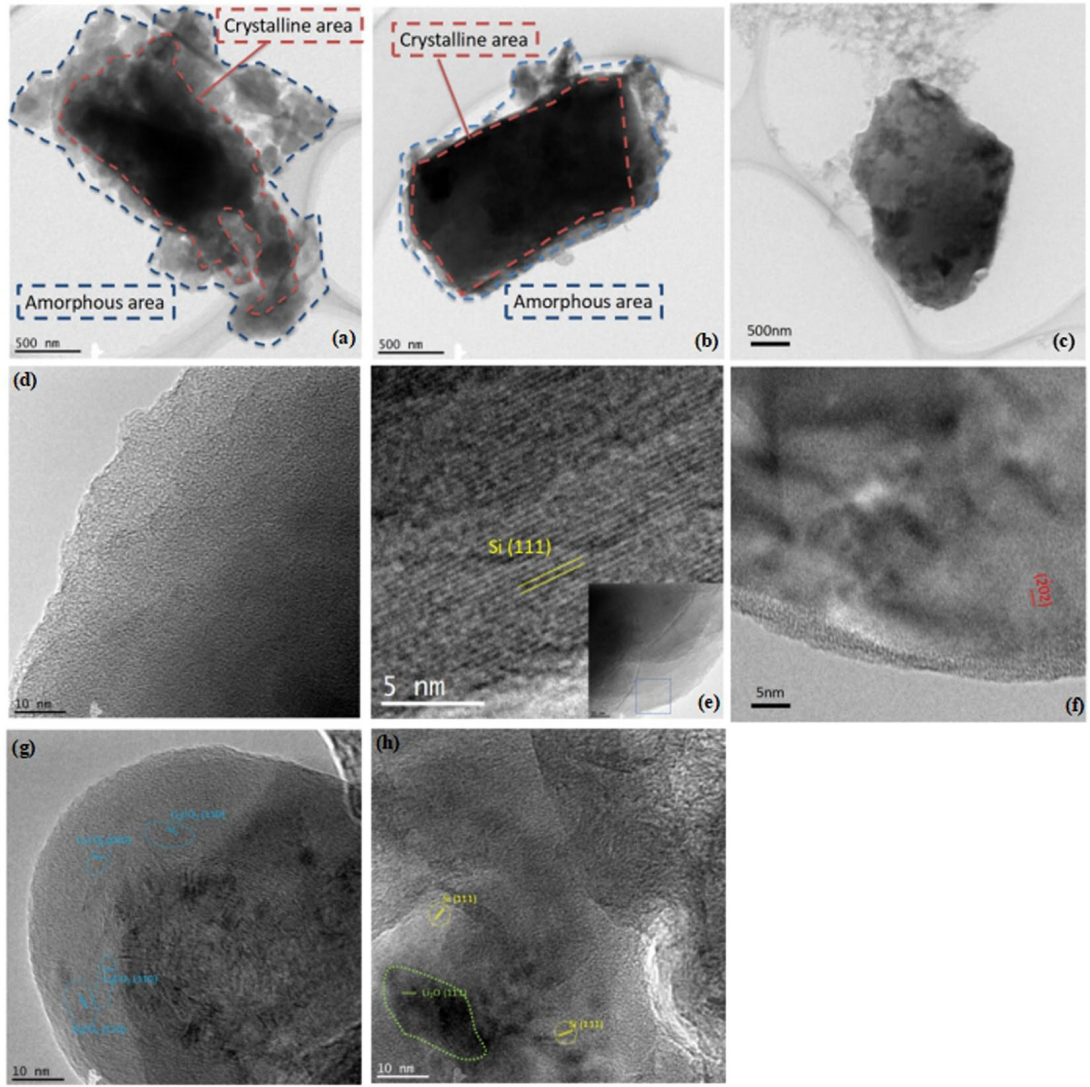
TEM images of silicon particles and SEI layer in silicon particles pre-doped under pressure (**a**,**d**,**g**), pre-doped without pressure (**b**,**e**,**h**), and pristine (**c**,**f**).

Figure 4 shows TEM images of the electrochemically pre-doped and pristine silicon particles. The pristine silicon had a clean surface and a thin amorphous edge (Fig. 4c,f), whereas the surface composition of the pre-doped silicon samples depended on the pressure. After the silicon anode was pre-doped by discharging under pressure, the amorphous layer thickened because the crystallinity of the silicon was destroyed by lithium intercalation and SEI formation (Fig. 4a). The samples electrochemically pre-doped under pressure had a sea-island structure of Li_2_CO_3_ particles with their observed lattice planes and certain island structure distributed in the SEI layer (Fig. 4g and Supplementary Fig. 1a). There was no carbon in the pristine silicon anode, suggesting that the CO_3_^2−^ originated from the reductive reaction of the electrolyte.

The TEM images of the sample electrochemically pre-doped without pressure (Fig. 4b,h and Supplementary Fig. 1c) show the lattice planes in the SEI layer converted from Li_2_CO_3_ to Li_2_O and silicon, and the SEI is much thinner than that formed under pressure. Because of insufficient doping in a limited doping time, no further reductive reaction of the electrolyte with Li_2_O to generate Li_2_CO_3_ occurred, and the silicon doping remained low with no further lithiation. Lattice fringes were also observed for the particles of the sample electrochemically pre-doped without pressure (Fig. 4e), which is consistent with the explanation.

**Figure 5:**
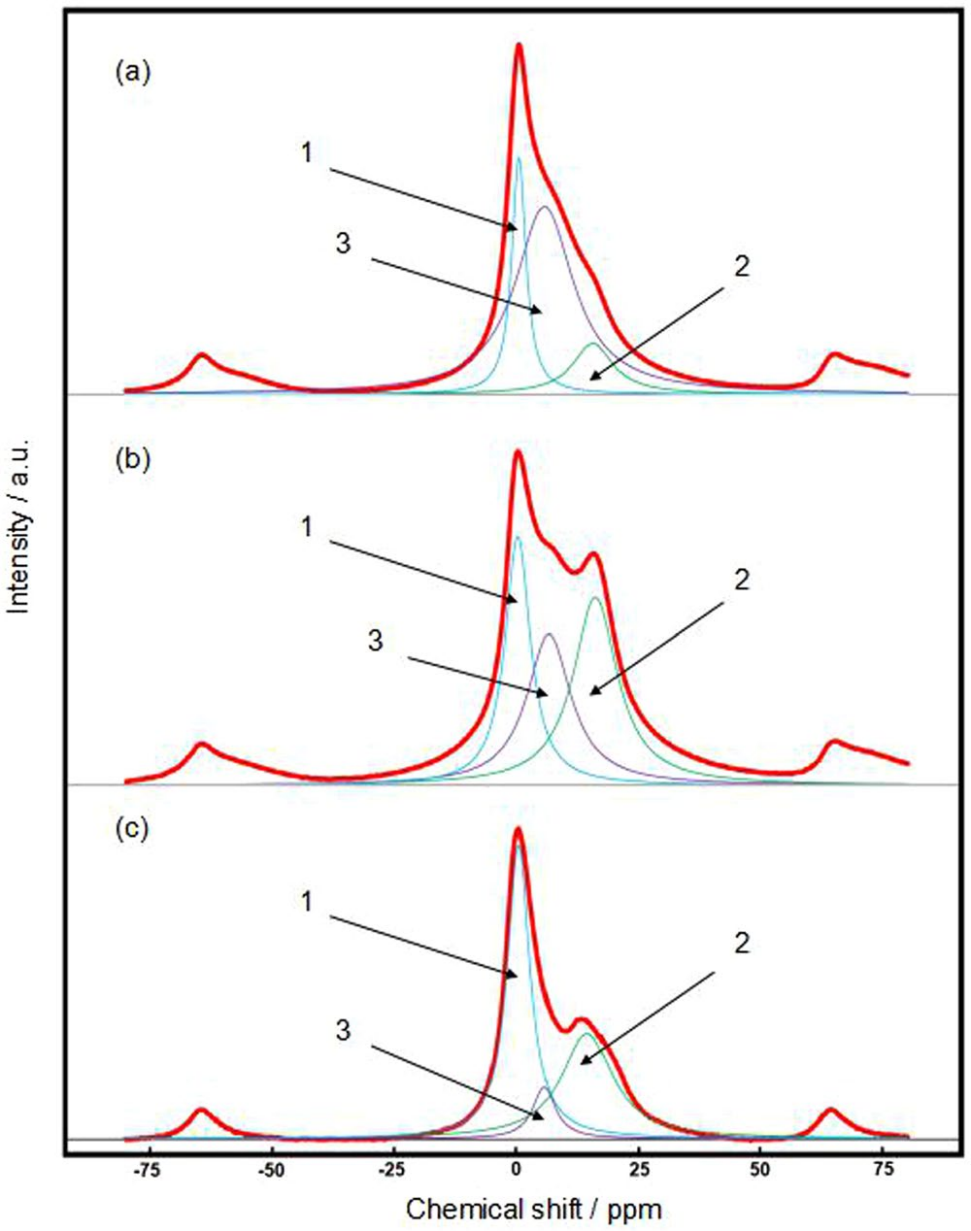
Solid state ^7^Li MAS NMR spectra of silicon samples electrochemically pre-doped under pressure (**a**), electrochemically pre-doped without pressure, (**b**) and contact pre-doped with lithium metal (**c**).

Solid state ^7^Li MAS NMR experiments were performed on silicon anodes electrochemically pre-doped under pressure, without pressure, and contact pre-doped with the same doping level (2 mol/mol). ^7^Li NMR spectra of the all silicon samples showed three resonances at 0.3, 6.0, and 18.0 ppm from overlapping single peaks, which were separated and fitted based on literature data^11^. The integral intensity of the higher binding number Li_*x*_Si complex (2.3 < *x* < 3.8) decreased, whereas that of the lower binding number Li_*x*_Si complex (*x* < 1) increased in the order of the samples electrochemically pre-doped under pressure, without pressure, and contact-doped (Fig. 5 and Table 1). In the contact-doped sample, large amounts of composite Li_*x*_Si complexes with *x* < 1 and 1.7 < *x* < 2.3 were formed, which indicated that in the main reactions, one silicon atom does not combine with more than two lithium ions (Fig. 5a and Table 1). Si K edge EXAFS measurements of the samples contact-doped, electrochemically pre-doped without pressure, and electrochemically pre-doped under pressure also showed similar results as given in Supplementary Fig. 2. One explanation may be that the non-uniform contact of the silicon surface with lithium causes a heterogeneous lithium ion concentration distribution over the interface. In the sample that was electrochemically pre-doped without pressure, the amount of the Li_*x*_Si complex with 0 < *x* < 2.3 was higher than in the contact sample (Fig. 5b and Table 1). In the sample that was electrochemically pre-doped under pressure, the amount of the Li_*x*_Si complex with 2.3 < *x* < 3.8 was the highest, and that with 1.7 < *x* < 2.3 was the lowest (Fig. 5a and Table 1). The most significant difference of the sample electrochemically pre-doped under pressure compared with other samples is that the ratio of highly doped one (2.3 < x < 3.8 Li_*x*_Si) and that of slightly doped one (x < 1 Li_*x*_Si) are larger than that of moderately doped one (1.7 < *x* < 2.3 Li_*x*_Si), causing bipolarization of the doped products. These results suggest that several lithium ions bound to one silicon atom under pressure. To explain this result, the following two reactions between silicon and lithium with different stoichiometries were considered.

1 $$7{\rm{Li}}+{\rm{3Si}}\to {{\rm{Li}}}_{7}{{\rm{Si}}}_{3}({{\rm{Li}}}_{2.33}{\rm{Si}})$$

2 $$15{\rm{Li}}+4{\rm{Si}}\to {{\rm{Li}}}_{15}{{\rm{Si}}}_{4}({{\rm{Li}}}_{3.75}{\rm{Si}})$$

**Table 1:** Solid state ^7^Li MAS NMR data for Li_x_Si components in pre-doped silicon electrodes.

Component Li_x_Si	Chemical shift/ppm	Integral intensity/%		
		Under pressure	Without pressure	Contact
x < 1	0.6	24	31	41
1.7 < x < 2.3	15.7	16	44	50
2.3 < x < 3.8	5.8	60	25	9

According to Key *et al*., reaction (1) reaches the theoretical capacity of 2340 mA h/g^16^_._ The doping process for the silicon anodes that were contact-doped and electrochemically pre-doped without pressure stopped in the range of 0 < *x* < 2.3, indicating that the amount of pre-doped lithium was limited to half the silicon theoretical capacity (molar weight). Pressure appeared to promote the reaction (2) to take place even in the theoretical capacity of reaction (1), increasing the amount of pre-doped lithium Li_15_Si_4_ to 60% (Table 1). Additionally, the ability of silicon to combine with several lithium ions during pre-doping explains why electrochemically pre-doping under pressure occurred continuously and approached the theoretical capacity in the charge curve (Fig. 2). The higher amount of doped lithium in the silicon under pressure than that without pressure was also demonstrated in the Si *K* absorption edge XAFS results (Supplementary Fig. 3).

**Figure 6:**
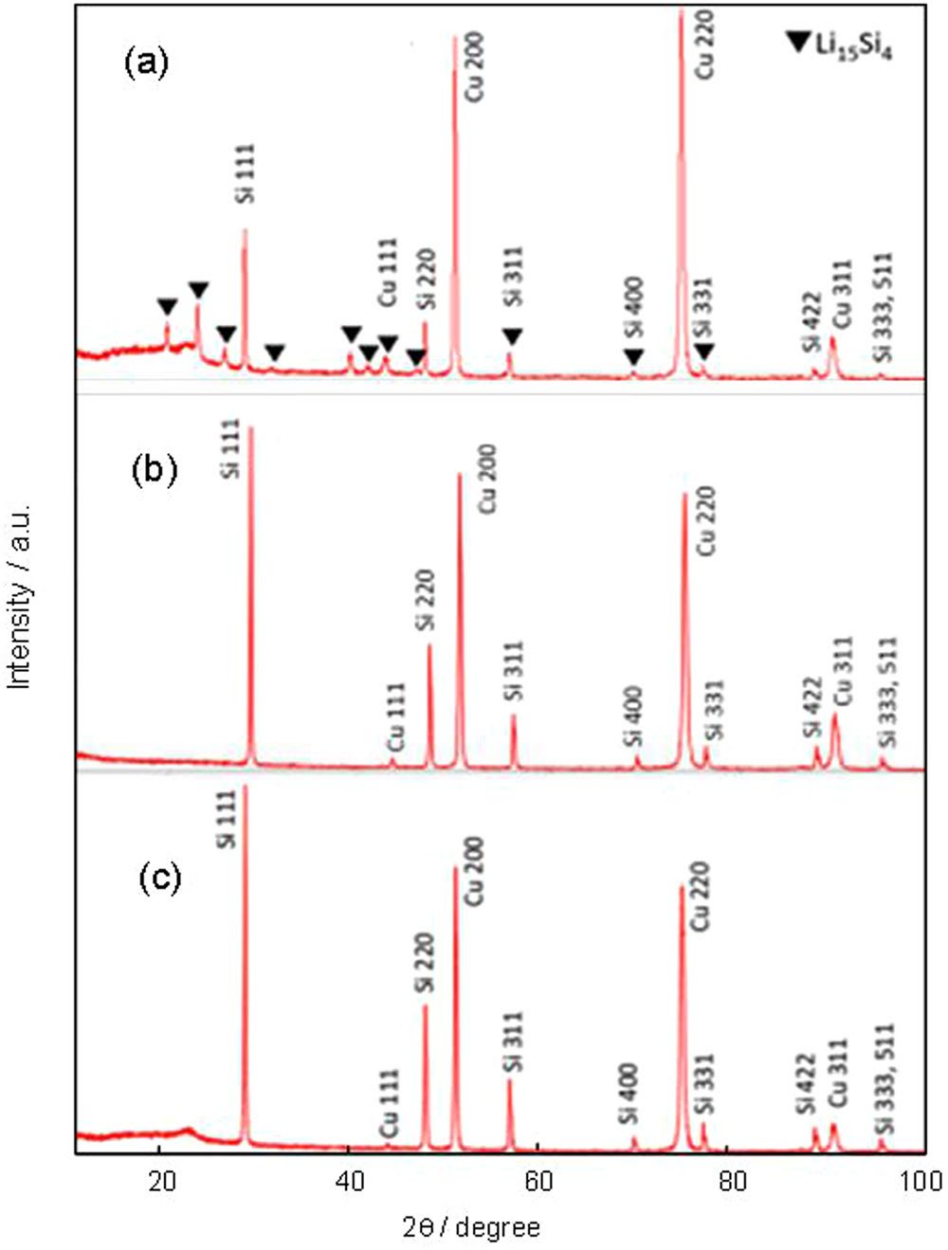
XRD patterns of pre-doped silicon electrodes electrochemically pre-doped under pressure (**a**), electrochemically pre-doped without pressure (**b**), and pristine silicon anode (**c**).

XRD was also used to explore the difference among the samples, electrochemically pre-doped under pressure, without pressure and pristine silicon anode. The XRD patterns were consistent with the evolution of the NMR peaks (Fig. 6). In the pristine silicon anode sample, in addition to the silicon peaks, only a crystalline phase signal for copper from the electrode foil was detected (Fig. 6c). A similar pattern was observed for the sample that was electrochemically pre-doped without pressure (Fig. 6b). The lack of signals detected for doped lithium shows its negligible presence, and the difference between the two patterns was negligible. In contrast, the pattern for the sample electrochemically pre-doped under pressure contained several small signals for Li_15_Si_4_ (Fig. 6a).

**Figure 7:**
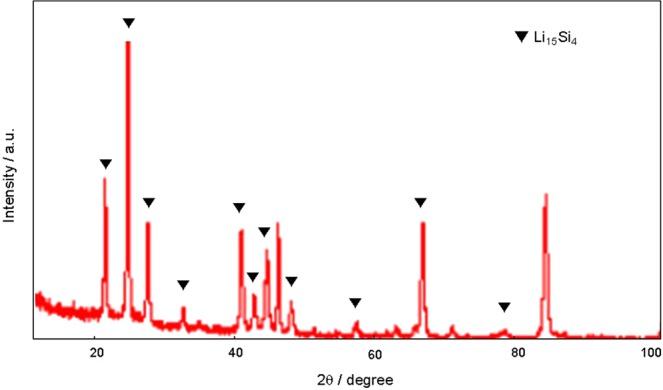
XRD patterns of the silicon electrode electrochemically pre-doped under pressure after five charge/discharge cycles.

Finally, we assembled a full cell composed of an anode of silicon electrochemically pre-doped under pressure on stainless foil and a cathode of LiNCM, and performed five charge-discharge cycles. The cell was disassembled in the charged state and a sample was analyzed by XRD. The pattern showed that the Si–Li complex in the full cell after operation was Li_15_Si_4_, even after five cycles (Fig. 7). This indicates that Li_15_Si_4_ is the stable state of lithium during the charging and discharging process in the silicon anode electrochemically pre-doped under pressure.

## Conclusion

We performed electrochemical pre-doping under pressure to improve the properties of a silicon anode. We characterized the SEI layer formed on the silicon anodes by pre-doping, and revealed that pressure efficiently promoted SEI formation associated with Li_2_CO_3_ formation, and increased lithium insertion lead to one silicon particle tends to bind with more than two lithium particles. Li_15_Si_4_ was stable in a full cell after five charge–discharge cycles, suggesting that the silicon anode may have a long cycle life. The improved cycling performance and higher energy density was due to the formation of a complete SEI and high electrochemical lithium pre-doping under pressure. However, the electrochemical pre-doping under pressure requires further optimization to achieve precise control. The potential application of the method in combination with LIBs and other new batteries should also be further studied.

## Methods

### Materials and pre-doping

The silicon particles were commercially available silicon fine powder (Si > 99.5%, Silgrain e-Si 409, Elkem). The silicon electrode was prepared from an aqueous slurry of pure silicon particles, conductive additive (Denka Black Li-435, Denka), and binder (polyacrylic acid, Mw = 450,000, Sigma-Aldrich) in a weight ratio of 70:15:15. The slurry was agitated with a planetary mixer and homogenizer mixer (20,000 rpm, 8 min), and then coated on copper foil with a knife-over-roll coater and dried at 80 °C for 10 min. To reduce the weight, the copper foil was replaced with stainless steel foil in subsequent experiments. The foil was cut and connected with a nickel tab by ultrasonic welding, and then dried at 105 °C under vacuum for 2 h before use. The 45-μm-thick silicon electrode had a density of 0.8 g/cm^3^.

The film-packed cells were sealed under vacuum, and consisted of a stacked silicon electrode, lithium-coated copper foil, a separator film, and an appropriate amount of electrolyte solution of 12.1 wt % LiPF_6_ in a mixture of ethylene carbonate (24.0 wt %), diethyl carbonate (55.9 wt %), and fluoroethylene carbonate (8.0 wt %). Electrochemical pre-doping under pressure and without pressure was performed with a bipotentiostat/galvanostat (Wave Driver 20, Pine Research) in a voltage range from open circuit voltage (~3.2 V) to 10 mV at a current value of 0.044 mA/cm^2^ at 25 °C.

Electrochemical pre-doping under pressure was carried out by pressing at 50 kPa with two glass plates and controlled by the elasticity of a spring.

We also prepared pre-doped samples by the contact process for comparison, in which a silicon electrode and a 10.7-μm-thick lithium foil were brought into direct contact and infused with an appropriate amount of electrolyte for 36 h at 25 °C. The amount of lithium foil was back calculated according to number of moles of lithium equal to the amount of charge equivalent to the set capacity density and sampling after confirming absorption and disappearance of the lithium foil.

The all pre-doped silicon electrodes were washed with hydrofluoro ether and dried under an argon atmosphere before measurements.

### Characterization

In our electrochemical experiments and characterization such as TEM, NMR and XRD, the capacity density of silicon anode pre-doping was set to value of 2.2 mA h/cm^2^, this was equal to 2500 mA h/g-Si.

TEM observations were carried out with an analytical field emission transmission electron microscope (Tecnai G2F20, FEI) with an accelerating voltage of 200 kV. Samples were taken from the disassembled coin cells in a glove box, transferred to the microscope without air exposure, and analyzed by using the powder dispersion method.

Solid state ^7^Li (MAS) NMR spectroscopy of the composition of pre-doped silicon anodes was conducted with an NMR spectrometer (AVANCE III 600, Bruker). The samples were transferred to the chamber under an argon atmosphere and measured by the single-pulse method at a frequency of 233.2 MHz. The scans were taken with a 4 mm diameter probe, a width of 1.2 μs (30° pulse), and a spinning speed of 15 kHz at room temperature. LiCoO_2_ (−0.5 ppm) was used as the standard to calibrate the chemical shifts after 1200 scans. The composition of the Si–Li complex was separated and determined by integrating the NMR peak area. The peaks were assigned according to assignments in the literature of other Si–Li complexes in silicon anodes^2^. The lithium 1 s spectrum contained peaks for Li_12_Si_7_ at 18.5 ppm, Li_7_Si_3_ at 16.5 ppm, Li_13_Si_4_ at 11.5 ppm, and Li_15_Si_4_ at 6.0 ppm.

XRD patterns were obtained with an X-ray diffractometer (Smart Lab 9 kW, Rigaku Corporation) at 45 kV and 200 mA.

All sample preparation and measurements for ^7^Li MAS NMR and XRD were performed under an argon atmosphere.

### Electrochemical measurements

The electrochemical properties of the pre-doped silicon electrodes and pristine silicon electrodes as reference were measured in rebuilt CR2032 coin cells, which were assembled in an argon glovebox with a lithium disk (300 μm thick, 13 mm diameter) as the counter electrode and a polyolefin microporous film as the separator, with 0.1 mL appropriate amount of an electrolyte solution of 1 M LiPF_6_ in a mixture of ethylene carbonate and diethyl carbonate (30/70 vol %). The charge and discharge properties were measured during galvanostatic cycling with a charge–discharge test system (TOSCAT-3100, TOYO SYSTEM Co., Ltd.) at a constant current of 0.44 mA/cm^2^ and voltage range of 0.001–3.5 V.

The charge capacity of the silicon anode in the first cycle indicated that the amount of intercalated removable free lithium, besides that consumed to form the SEI. The total of these two amounts of lithium was the pre-doping amount of lithium that was proportional to the first charge capacity because the lithium consumed to form the SEI was quantified according to the material. Additionally, the second and subsequent charge capacities indicated that the cycle performance, which corresponded to the incomplete formation of the SEI in the pre-doping process, caused the degradation and poor round Coulomb efficiency of the electrode during the cycles.

The properties of the pre-doped silicon electrodes and pristine silicon electrodes as reference were estimated with full cells using the conventional LIB cathodes. A LiNiCoMnO_2_ (LiNCM, Ni:Co:Mn = 3:2:3, Beijing Easpring Technology Co., Ltd.) cathode was prepared without any pressing process from an *N*-methyl pyrrolidone slurry consist of 89 wt % LiNCM, 6 wt % acetylene black (Denka Black Li-435, Denka), and 5 wt % binder (polyvinylidene fluoride). The density was measured at 1.65 g/cm^3^.

## Supplementary information

Supplementary Information.

## Data Availability

The data for the plots in this work and other findings from this study are available from the corresponding author upon reasonable request.
