# Supplementary material for: High-energy, Long-cycle-life Secondary Battery with Electrochemically Pre-doped Silicon Anode
Source: Sci Rep. 2020 Feb 21;10:3208. doi: 10.1038/s41598-020-59913-4 (PMC7035378; doi:10.1038/s41598-020-59913-4)
Supplement: Supplementary file 1 — Supplementary Information. [file 41598_2020_59913_MOESM1_ESM.pdf]

SUPPLEMENTARY INFORMATION

In the format provided by the authors and unedited.

High-energy, Long-cycle-life Secondary Battery with Electrochemically Pre-doped Silicon Anode

Ying Wang[1], Masaharu Satoh[1]*, Masazumi Arao[2], Masashi Matsumoto[2], Hideto Imai[2] and Hiroshi Nishihara[1]*

[1]Department of Chemistry, School of Science, The University of Tokyo, 7-3-1 Hongo, Bunkyo-ku, Tokyo 113-0033, Japan. [2]Analysis Platform Department, NISSAN ARC, LTD., 1 Natsushima-cho, 79-5 Tokiwadai, Yokosuka 237-0061, Japan. *e-mail: m-satoh@chem.s.u-tokyo.ac.jp; nisihara@chem.s.u-tokyo.ac.jp

Supplementary Table 1. Coordination number of silicon evaluated from the Si K edge EXAFS spectra in Supplementary Figure 2.

Sample	Shell	Coordination number	Bond distance (Å)	(σ^2) (Å^2)	R-factor
Pristine Si	Si-Si	4.0	2.35	0.0015	0.00212
Without pressure	Si-Si	2.3	2.35	0.0013	0.00414
Pressure	Si-Si	1.0	2.35	0.0015	0.07876

Supplementary Table 2. Round Coulomb efficiency value of full cells with a LiNCM cathode and various silicon anodes in Supplementary Figure 4.

Cycle number	Pristine Si	Contact	Without pressure	Under pressure
1	71.9	98.6	97.4	100
10	96.9	99.3	98.8	100
20	97.6	99.5	99.0	99.7
35	96.7	99.4	98.8	99.6
50	98.4	99.1	99.4	99.5

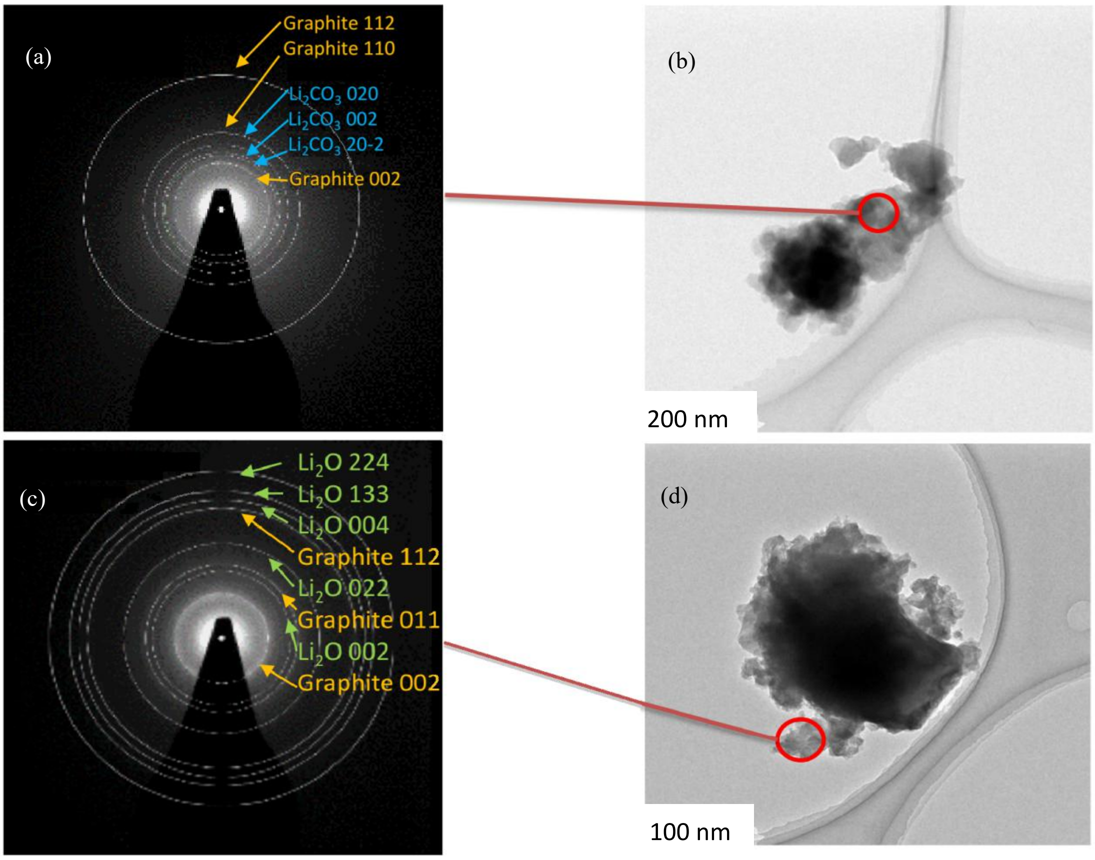

Supplementary Figure 1 | Selected area electron diffraction (SAED) patterns for silicon particles pre-doped under pressure (a), (b) and without pressure (c), (d). According to diffraction rings and diffraction spots, they showed graphite and Li_2CO_3 in pre-doped sample under pressure and graphite and Li_2O in pre-doped sample without pressure, respectively.

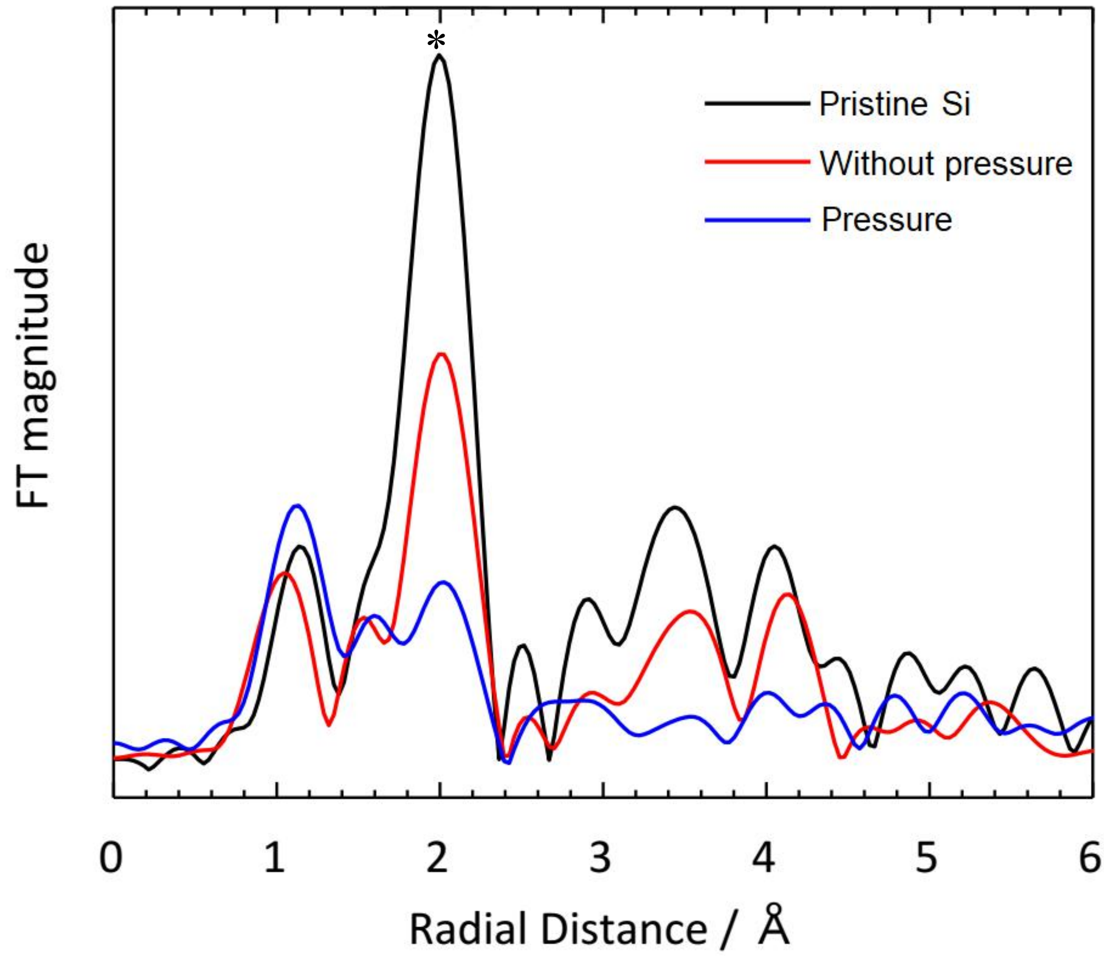

Supplementary Figure 2 | Si K edge EXAFS spectra. The intensity of Si-Si bonding peak (labeled with an asterisk,*) was reduced with the intercalation of lithium. The coordination number of silicon evaluated from the spectra is listed in Supplementary Table 1.

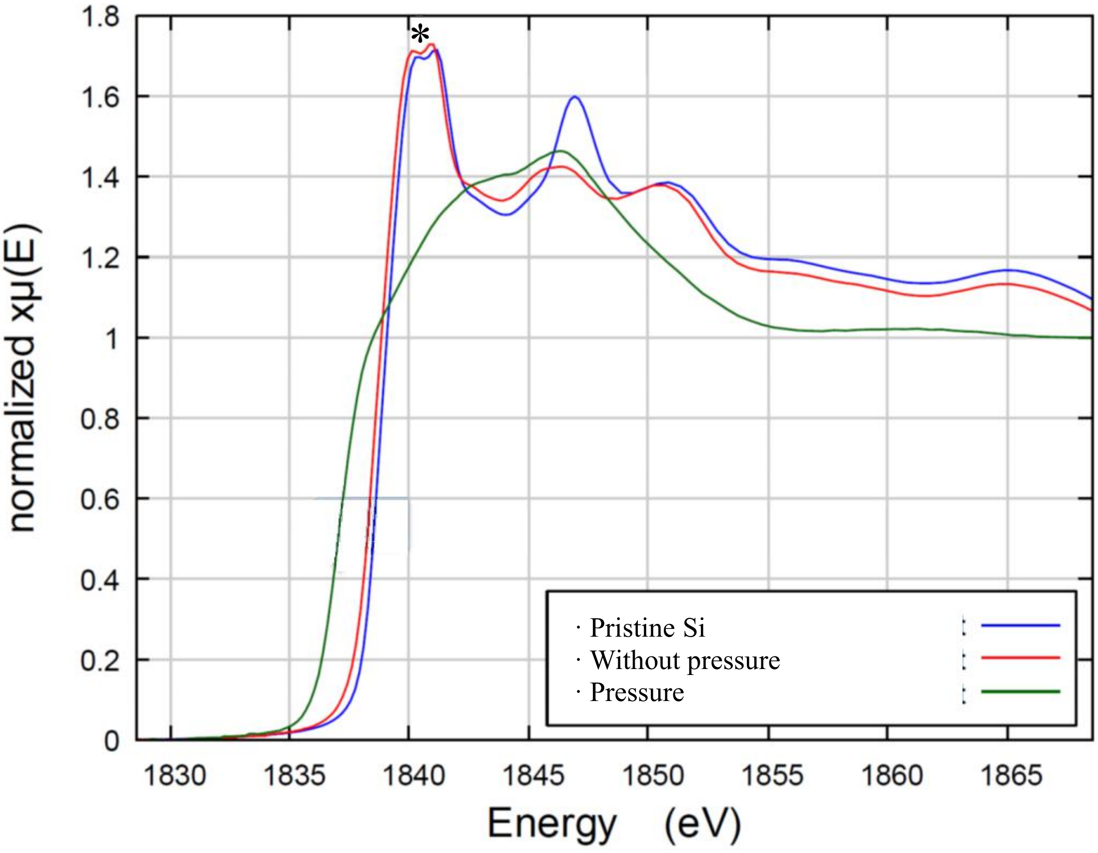

Supplementary Figure 3 | Si K absorption edge XAFS spectra. Generation of amorphous phase Li_xSi tended to shift the line to lower energy. In the sample under pressure, the peak of silicon (labeled with an asterisk, *) disappeared.

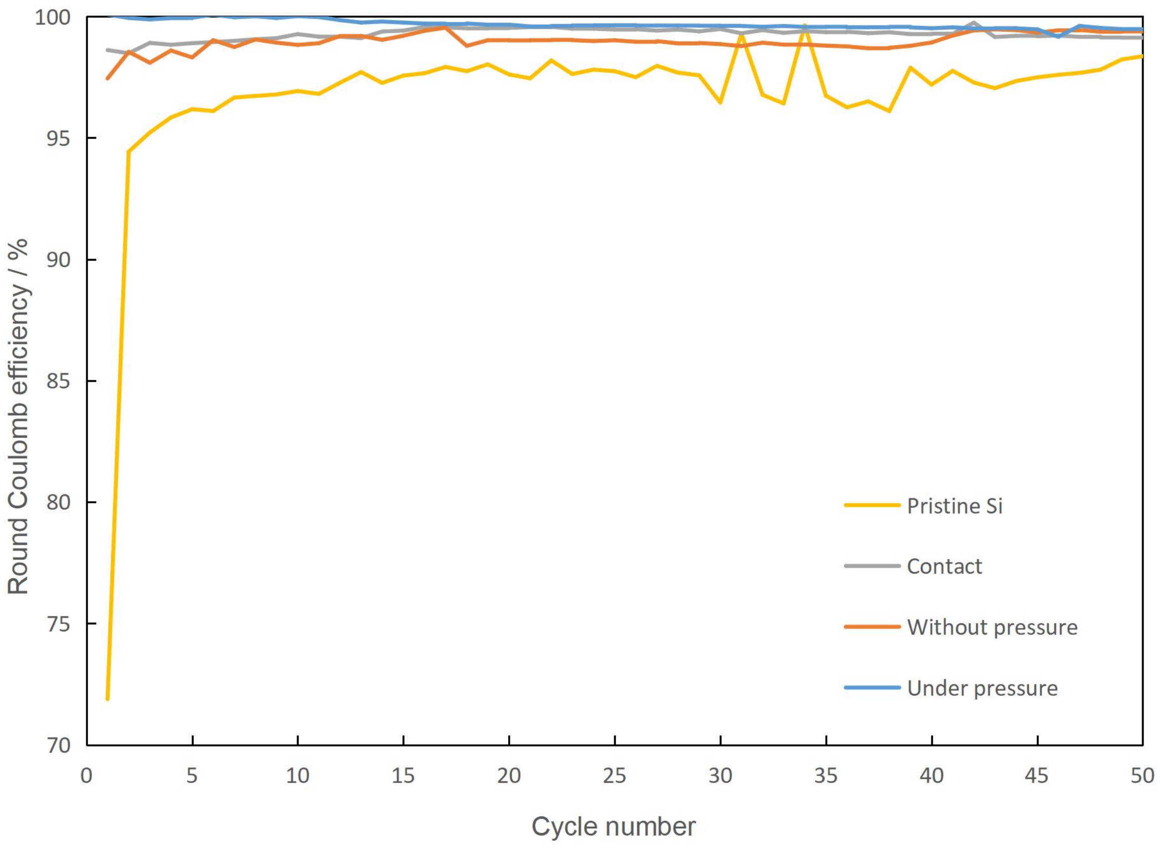

Supplementary Figure 4 | Round Coulomb efficiency of full cells with a LiNCM cathode and various silicon anodes showed in Fig. 3.
